# Editorial: Extracellular matrix modifications in development and disease

**DOI:** 10.3389/fcell.2025.1562296

**Published:** 2025-02-06

**Authors:** Karin Pfisterer, Michael Mildner, Maddy Parsons

**Affiliations:** ^1^ Department of Dermatology, Medical University of Vienna, Vienna, Austria; ^2^ Randall Centre for Cell and Molecular Biophysics, King’s College London, London, United Kingdom

**Keywords:** extracellular matrix (ECM), quantitative and qualitative ECM modifications, mathematic modelling, mechanobiology, ageing, cancer, fibrosis, development

The architecture and mechanical properties of the extracellular matrix (ECM) are crucial in shaping cell identity and phenotype as they influence transcription, differentiation, growth, morphology, motility, metabolism, and viability. Specialized cell types, such as fibroblasts, play a pivotal role in the biosynthesis and organization of ECM proteins in a tissue-specific manner, which is essential for development, homeostasis, and tissue repair. Alterations in ECM composition, structure, and mechanical properties are frequently observed in various diseases, including tissue fibrosis, autoimmune and inflammatory conditions, and cancer. Dysregulated ECM is a key driver of these conditions, making it a significant focus of research aimed at uncovering novel therapeutic pathways.

The composition, structure, and mechanical properties of the ECM in the context of development and disease have received increasing attention in recent years. This has led to the development of new probes, *in vitro* and *in vivo* models, high-resolution and *in situ* imaging methods, bioanalytical techniques, computational tools, and *in silico* modeling. These approaches are complemented by single-cell and spatial omics technologies to further profile transcriptional, proteomic and metabolic cell “states” associated with specific ECM phenotypes. These innovations have greatly advanced our understanding of the biological processes and mechanisms involved in ECM deposition, crosslinking, organization, and turnover in health and disease. Gaining a deeper understanding of the dynamic functional interplay between cells and their surrounding ECM is a pressing research challenge that demands multidisciplinary approaches to unravel and facilitate the development of new therapies.

This Research Topic brings together a multidisciplinary collection of studies focused on advancing our understanding of the diverse roles of the ECM in health and disease. The topics covered include current mathematical models to investigate ECM-cell interactions (Crossley et al.; Keijzer et al.), the role of ECM modifications in various diseases (Jahin et al.; Direder et al.; Guerrero-Barberà et al.; Töpfer and Holz) and in aging (Ulldemolins et al.).

Crossley et al. provided an overview of the structural components of the ECM, briefly describing its characteristics and function before deep-diving into a comprehensive description of currently available methods to model ECM dynamics mathematically. The authors compared two main approaches, the continuum model, which describes the tissue components as a whole using ordinary or partial differential equations, and the discrete agent-based model, in which the tissue components can be simulated individually, including lattice-based and -free approaches and the Cellular Potts model. The concepts of hybrid models incorporating multiple approaches and mechanical models were also discussed before addressing the advantages and limitations regarding the applicability of current mathematical models to the study of tissue mechanics, cell-matrix interactions and cell migration *in silico*.

Keijzer et al. developed a novel hybrid model that integrates a Cellular Potts model to describe shape changes in individual cells, a molecular dynamics model to simulate the dynamics of fibrous ECM networks under cell-generated forces, and an ordinary differential equation model to describe the dynamics of mechanosensitive focal adhesions. This computational approach captures both the influence of the ECM on cell morphology and orientation and the effects of cell contraction on the ECM structure. The authors incorporated focal adhesion turnover and stability in response to mechanical tension due to cell pulling on the ECM or structural and physical ECM properties such as fiber crosslinking, alignment, deformation, and stiffness. Thus, this hybrid model represents a powerful predictive tool for *in silico* investigations of cell-ECM interactions and reciprocal force generation.

Jahin et al. investigated the role of ECM stiffening in breast cancer spheroid behavior. Combining biomechanically tuned collagen I matrices and advanced 3D imaging, the authors demonstrated that two different breast cancer cell lines show increased YAP and ERK1/2 activity in response to increased matrix stiffness. Activation of both regulators is higher in cells contacting the ECM at the periphery of the spheroids compared to those in the center of the cell mass. Further analysis identified the α2β1 collagen-binding integrins as key mechanosensitive receptors in the transmission of stiffer ECM to signaling responses. Contrary to some previous reports, however, the authors demonstrated that this increased stiffness-dependent signaling correlates with lower proliferation, lower ECM alignment and reduced invasive potential of cells from the spheroid. These findings support the notion that stiffer ECM, as seen in several cancer types, is sufficient to support pro-tumorigenic signaling but that functional consequences of this signaling require contributions from additional cell types within the tumor microenvironment.

Direder et al. conducted an investigation into the cellular composition and gene regulatory mechanisms underlying idiopathic subglottic stenosis (ISGS), a rare fibrotic disease of the upper trachea, utilizing single-cell RNA sequencing. Their analysis identified fibroblasts and Schwann cells, glial cells of the peripheral nervous system, that exhibited a distinct profibrotic phenotype. Furthermore, the study revealed a marked increase in plasma cell populations in ISGS. These findings provide novel insights into the pathogenesis of ISGS, potentially advancing the development of innovative diagnostic and therapeutic strategies.

Guerrero-Barberà et al. comprehensively reviewed the role of the abundant ECM protein fibronectin in cancer progression. From transcriptional regulation and biosynthesis to cell binding, scaffolding and tumorigenic properties, the authors highlight critical functions for this molecule across different regions in the tumor microenvironment. They further explored the potential for future domain-specific therapeutic targeting of fibronectin, both directly and in conjunction with integrin antagonists or immunotherapy.

Töpfer and Holz provided a concise overview of nidogen, a multifunctional glycoprotein, highlighting its role in basement membrane integrity, morphogenesis, and neuronal plasticity. This review emphasizes nidogen’s critical function as a linker molecule within the extracellular matrix, effectively connecting different components. Additionally, the authors explored the specific roles of nidogen in cell adhesion, migration, and signaling.

Ulldemolins et al. investigated how aging affects the mechanical properties of lung tissue using a mouse model. They measured age-related changes in ECM composition and biomechanical properties in decellularized lungs from young and aged mice. Comparing stiffness, viscosity, and adhesion forces in non-inflated and inflated lungs via atomic force microscopy, they found reduced stiffness in aged non-inflated lungs but increased stiffness at functionally inflated volumes. In contrast to prior studies, which focused on non-inflated lungs, this work provides novel insights into lung mechanics under physiological stretch conditions.

